# Cardiovascular safety of sitagliptin in patients with type 2 diabetes mellitus: a pooled analysis

**DOI:** 10.1186/1475-2840-12-3

**Published:** 2013-01-03

**Authors:** Samuel S Engel, Gregory T Golm, Deborah Shapiro, Michael J Davies, Keith D Kaufman, Barry J Goldstein

**Affiliations:** 1 Merck Sharp & Dohme Corp, Whitehouse Station, NJ, USA

## Abstract

**Objective:**

To compare the incidence of cardiovascular events and mortality in patients with type 2 diabetes mellitus treated with sitagliptin or non-sitagliptin comparators.

**Methods:**

A *post hoc* assessment of cardiovascular safety in 14,611 patients was performed by pooling data from 25 double-blind studies, which randomised patients at baseline to sitagliptin 100 mg/day or a non-sitagliptin comparator (i.e., non-exposed). Included studies were limited to those at least 12 weeks in duration (range: 12 to 104 weeks). Patient-level data were used in this analysis of major adverse cardiovascular events (MACE) including ischaemic events and cardiovascular deaths. Analyses were performed in three cohorts: the entire 25-study cohort, the cohort from placebo-controlled portions of studies (n=19), and the cohort from studies comparing sitagliptin to a sulphonylurea (n=3).

**Results:**

In the entire cohort analysis, 78 patients had at least 1 reported MACE-related event, with 40 in the sitagliptin group and 38 in the non-exposed group. The exposure-adjusted incidence rate was 0.65 per 100 patient-years in the sitagliptin group and 0.74 in the non-exposed group (incidence rate ratio = 0.83 [95% confidence interval (CI): 0.53, 1.30]). In the analysis comparing sitagliptin to placebo, the exposure-adjusted incidence rate was 0.80 per 100-patient-years with sitagliptin and 0.76 with placebo (incidence rate ratio = 1.01 [95% CI: 0.55, 1.86]). In the analysis comparing sitagliptin to sulphonylurea, the exposure-adjusted incidence rate was 0.00 per 100 patient-years with sitagliptin and 0.86 with sulphonylurea (incidence rate ratio = 0.00 [95% CI: 0.00, 0.31]).

**Conclusion:**

A pooled analysis of 25 randomised clinical trials does not indicate that treatment with sitagliptin increases cardiovascular risk in patients with type 2 diabetes mellitus. In a subanalysis, a higher rate of cardiovascular-related events was associated with sulphonylurea relative to sitagliptin.

## Introduction

Type 2 diabetes mellitus is associated with an increased risk of cardiovascular disease and mortality [1,2]. Cardiovascular events account for approximately 70% of deaths in older patients with type 2 diabetes mellitus [3]. Furthermore, short- and long-term survival following a myocardial infarction is lower in patients with type 2 diabetes mellitus compared to those without [4-8]. In a prospective observational study, the risk of a subsequent myocardial infarction in patients with pre-existing diabetes was found to be comparable to patients with pre-existing coronary disease [9]. These and other data have supported the concept that type 2 diabetes mellitus is considered a coronary heart disease risk equivalent; treatment guidelines for lipid management for patients with type 2 diabetes parallel those for patients with prior coronary events [10]. Thus, prevention of cardiovascular disease is a major clinical challenge in treating patients with type 2 diabetes mellitus.

The potential role of antihyperglycaemic medications in the development and/or progression of cardiovascular disease has received increasing attention, related in large part to the observation that rosiglitazone was associated with an increased incidence of cardiovascular events [11-13]. As a reflection of the heightened concern regarding the intrinsic effects of antihyperglycaemic agents on cardiovascular safety, in 2008, the FDA instituted requirements for the assessment of cardiovascular safety as a key component of the clinical development programs for new antihyperglycaemic agents [14].

DPP-4 inhibitors are a newer class of antihyperglycaemic therapy and improve glycaemic control by inhibiting the inactivation of the incretin hormones, glucagon-like peptide-1 (GLP-1) and glucose-dependent insulinotropic polypeptide [15]. Sitagliptin, the first agent approved in this class of antihyperglycaemic agents, was introduced for clinical use in 2006. To date, DPP-4 inhibitors (sitagliptin, saxagliptin, vildagliptin, linagliptin, and alogliptin) have not been shown to be associated with an increased risk of cardiovascular events [16-20]. In view of the increased focus on the effects of antihyperglycaemic agents on cardiovascular outcomes, the present analysis expanded upon a previous cardiovascular assessment of sitagliptin [16] by including results from recently completed sitagliptin trials.

## Methods

The present *post hoc* analysis used a pooled population (N = 14,611) drawn from all 25 multicenter, U.S. or multinational, double-blind, parallel-group studies conducted by Merck & Co., Inc., in which patients were randomised to receive sitagliptin 100 mg/day (n = 7,726) or a comparator (n = 6,885) for at least 12 weeks and up to 2 years (the duration of the longest studies) and for which results were available as of December 1, 2011 (complete study listing in Appendix I, Table 2). Each protocol was reviewed and approved by appropriate ethical review committees and authorities for each clinical site. All patients were to have provided written informed consent. The studies evaluated sitagliptin as monotherapy, initial combination therapy with either metformin or pioglitazone, or add-on combination therapy with other antihyperglycaemic agents including metformin, pioglitazone, a sulphonylurea (with and without metformin), insulin (with and without metformin), or metformin + rosiglitazone or pioglitazone. Patients not receiving sitagliptin (i.e., the non-exposed group) received placebo, metformin, pioglitazone, a sulphonylurea (with and without metformin), insulin (with and without metformin), or metformin + rosiglitazone or pioglitazone. From each contributing study, the pooling was conducted by including those portions that had parallel treatment groups with concurrent exposures to sitagliptin 100 mg/day (primarily administered as 100 mg once daily) or other treatments (either placebo or active comparator). Studies conducted only in Japan were excluded from all analyses; a lower starting dose of sitagliptin has been separately developed in Japan. The pooling excluded studies conducted in patients with moderate to severe renal insufficiency, because these patients received sitagliptin at doses less than 100 mg/day.

In each study, investigators were to report adverse events (serious and non-serious) that occurred during the conduct of the study, as well as serious adverse events occurring within 14 days following the last dose of blinded study drug. The present analysis used patient-level data from each study to assess the incidence rates of cardiovascular-related adverse events that occurred following initiation of double-blind study drug. Many studies in this analysis included open-label glycaemic rescue therapy, which was to have been initiated based upon progressively stricter, protocol-specified hyperglycaemia criteria. When initiated, glycaemic rescue therapy was added to the ongoing, blinded study medication to which patients had been randomised. The analysis in this pooled population includes all post-randomisation events reported to have occurred during a given study, including those events with onset after the initiation of glycaemic rescue therapy.

The primary outcome was major adverse cardiovascular events (MACE), which comprised ischaemic events and cardiovascular deaths (see Appendix II, Tables 3 and 4, for the definition of MACE used in the analysis). Analyses were performed in three cohorts: the entire 25-study cohort (sitagliptin vs. non-exposed), the cohort from placebo-controlled portions of studies (sitagliptin vs. placebo), and the cohort from studies comparing sitagliptin to a sulphonylurea (sitagliptin vs. sulphonylurea). The sitagliptin vs. placebo analysis was performed to eliminate any potential confounding effects from the various active comparators. The data were pooled from the placebo-controlled portions of 19 double-blind studies, which randomised patients at baseline to sitagliptin 100 mg/day (n = 5,236) or placebo (n = 4,548) for up to 1 year (Appendix I, Table 2). Since sulphonylureas have been associated with an increased risk for cardiovascular events relative to metformin in some, but not all, observational studies [21-26], the sitagliptin vs. sulphonylurea analysis was performed by pooling the three double-blind studies (P010, P024, P803), which randomised patients at baseline to sitagliptin 100 mg/day (n = 1,226) or a sulphonylurea (n = 1,225) for up to 2 years [27-30]. Two sensitivity analyses were performed to assess the robustness of the results from the primary comparisons of sitagliptin vs. non-exposed and sitagliptin vs. sulphonylurea. These analyses included all trials or portions of trials in which sitagliptin 100 mg or corresponding control were given in a blinded fashion, even if those treatments reflected a switch from the treatment given at randomization. The first sensitivity analysis extended the primary analysis of sitagliptin vs. non-exposed by adding all patients from two phase 2 dose-ranging studies (P010, P014) who initially received placebo or doses of sitagliptin less than 100 mg, but were subsequently switched to sitagliptin 100 mg. Only those events that occurred after the switch to sitagliptin 100 mg were counted in the analysis. The second sensitivity analysis extended the primary analysis of sitagliptin vs. sulphonylurea by adding the same patients from P010 who were added to the first sensitivity analysis, as well as all patients from a 104-week phase 3 study (P020) in which patients were randomised to receive sitagliptin or placebo for the first 24 weeks, with the placebo group switching to a sulphonylurea after Week 24. Only the Week 24 to Week 104 data from P020 were included in the second sensitivity analysis.

### Statistical Analyses

To account for potential differences between groups in duration of exposure to treatment, MACE was analyzed in terms of exposure-adjusted incidence rates (i.e., the number of patients with ≥1 event divided by the total patient-years of exposure). For patients who had an event, exposure was calculated as the time from the first dose of study medication at randomisation to the time that the first post-randomisation event occurred. For patients without an event, exposure was calculated as the time from the first dose to 14 days after the last dose. Exposure-adjusted incidence rate ratios (sitagliptin relative to comparator) and the associated 95% confidence intervals (CI) were calculated using an exact method for Poisson processes [31], stratified by study. Studies in which no events occurred were excluded from analyses using this approach. A sensitivity analysis was conducted using the Mantel-Haenszel method [32], which included studies with no events by use of a continuity correction factor. An additional sensitivity analysis was conducted using Cox regression. All analyses were performed using SAS Version 9.1.

## Results

At baseline, patients in the entire 25-study cohort (55% male) had an average age of 54 years (range: 19 to 91 years; 17% ≥65 years), a median duration of diabetes of 3.5 years, and a mean HbA_1c_ of 8.4% (Table 1). The cohort was 61% Caucasian, 18% Asian, and 6% Black. At baseline, 10% of patients had a history of cardiovascular disease, and 81% had additional cardiovascular risk factors besides type 2 diabetes mellitus and cardiovascular disease, including hypertension (53%), history of dyslipidaemia/hypercholesterolaemia (49%), and history of smoking (39%). There were no meaningful differences between groups in these baseline characteristics.

**Table 1:** Baseline characteristics of randomised patients

**Characteristic**	**Sitagliptin**	**Non-exposed**
	**(n = 7,726)**	**(n = 6,885)**
Age, years	54.0 ± 10.3	54.4 ± 10.5
Age ≥65 years, n (%)	1,261 (16)	1,185 (17)
Gender, n (%)		
Male	4,196 (54)	3,788 (55)
Female	3,530 (46)	3,097 (45)
Race, n (%)		
Caucasian	4,674 (60)	4,227 (61)
Black	427 (6)	384 (6)
Asian	1,436 (19)	1,227 (18)
Multiracial	462 (6)	427 (6)
Other or unknown	727 (9)	620 (9)
Ethnicity, Hispanic or Latino, n (%)	1,917 (25)	1,690 (25)
Body weight, kg	85.0 ± 19.6	85.8 ± 20.1
Body mass index, kg/m^2^	30.5 ± 5.7	30.7 ± 5.8
Duration of T2DM, years*	3.0	4.0
Distribution of duration of T2DM, n (%)^†^		
<5 years	4,535 (59)	4,002 (58)
≥5 and <10 years	1,864 (24)	1,690 (25)
≥10 years	1,316 (17)	1,188 (17)
HbA_1c_, %	8.4 ± 1.3	8.4 ± 1.3
HbA_1c_ distribution at baseline, n (%)		
HbA_1c_ <8%	3,190 (41)	2,924 (42)
HbA_1c_ ≥8 to <9%	2,258 (29)	1,931 (28)
HbA_1c_ ≥9%	2,264 (29)	2,016 (29)
History of CVD, n (%)	792 (10)	691 (10)
Proportion of patients with known CV risk factors other than T2DM and history of CVD, n (%)^††^	5,827 (81)	5,266 (82)
History of dyslipidaemia, n (%)	3,857 (50)	3,350 (49)
History of hypertension, n (%)	4,110 (53)	3,666 (53)
History of smoking, n (%)^††^	2,712 (38)	2,539 (39)

T2DM = type 2 diabetes mellitus, CV=cardiovascular, CVD = cardiovascular disease.

Data are expressed as mean (± standard deviation) or frequency (n [%]), unless otherwise indicated.

* Median.

^†^ Number of patients with unknown duration of diabetes was 11 in the sitagliptin group and 5 in the non-exposed group.

^††^ Denominator is 7,177 and 6,451, respectively because history of smoking was not routinely collected in all sitagliptin studies.

The mean exposure to study drug was greater in the sitagliptin group relative to the non-exposed group: 284 dosing days (range: 1 to 791) relative to 264 dosing days (range: 1 to 801), respectively. In the sitagliptin group, 2,457 (32%) patients were treated for at least 1 year, with 584 of these patients treated for 2 years; the corresponding numbers of patients were 1,775 (26%) and 470 in the non-exposed group. In this pooled analysis of studies 12 weeks to 2 years in duration, the proportions of patients discontinuing treatment were 27.2% in the sitagliptin group and 28.8% in the non-exposed group.

### MACE Analyses

#### Entire 25-study Cohort

**Figure 1:**
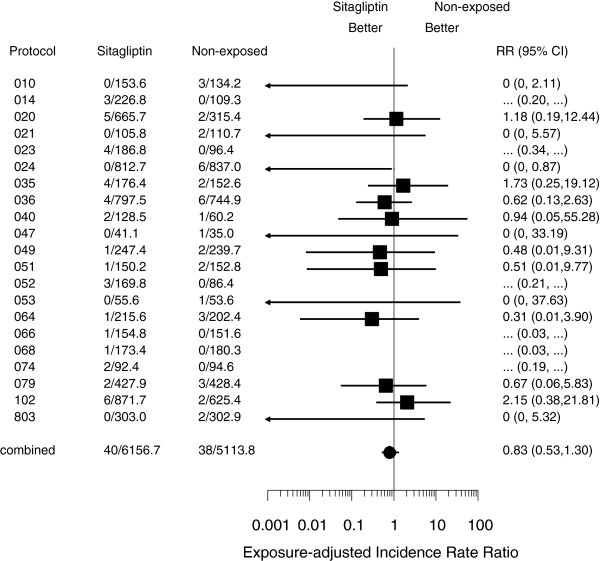
**Forest plot of custom MACE (Exposure-adjusted incidence rate ratios).** The second and third columns display the number of patients with ≥1 event/patient-years follow-up time. The estimates of rate ratio and 95% confidence intervals (CI) are calculated using exact method stratified by study. Studies with no events in both sitagliptin and non-exposed groups are excluded from the treatment comparison. RR denotes adjusted incidence rate ratio.

In the 25-study cohort, events occurred in 21 of the studies. After excluding the 4 studies with no events (P019, P061, P128, and P801; Appendix I, Table 2), 13,462 of the 14,611 patients contributed to the primary analysis using the exact method, and the cumulative patient exposure was 6,157 patient-years for the sitagliptin group and 5,114 patient-years for the non-exposed group. A total of 78 patients had at least one reported MACE-related event, with 40 in the sitagliptin group and 38 in the non-exposed group. The exposure-adjusted incidence rates per 100 patient-years were 0.65 in the sitagliptin group and 0.74 in the non-exposed group (adjusted incidence rate ratio = 0.83 [95% CI: 0.53, 1.30]) (Figure 1). For cardiovascular-related deaths, there were 12 in the sitagliptin group compared to 10 in the non-exposed group. The exposure-adjusted incidence rate for cardiovascular-related death was 0.25 per 100-patient-years for both the sitagliptin and non-exposed group (adjusted incidence rate ratio = 0.95 [95% CI: 0.40, 2.30]). In the sensitivity analysis that included all blinded exposure to sitagliptin 100 mg (n = 8,128 sitagliptin, n = 6,885 non-exposed), the cumulative patient exposure excluding studies with no events was 6,596 patient-years for the sitagliptin group and 5,114 patient-years for the non-exposed group. A total of 86 patients had at least one MACE-related event reported, with 48 in the sitagliptin group and 38 in the non-exposed group. The exposure-adjusted incidence rate was 0.73 per 100-patient-years in the sitagliptin group and 0.74 in the non-exposed group (adjusted incidence rate ratio = 0.87 [95% CI: 0.56, 1.35]).

#### Sitagliptin vs. Placebo

Baseline characteristics were similar between treatment arms (data not shown). The cumulative patient exposure was approximately 3,335 patient-years with sitagliptin and 2,922 patient-years with placebo. In this 19-study subanalysis, 24 patients in the sitagliptin group compared to 20 in the placebo group had at least one MACE-related event. The exposure-adjusted incidence rate was 0.80 per 100-patient-years with sitagliptin and 0.76 with placebo (adjusted incidence rate ratio = 1.01 [95% CI: 0.55, 1.86]). There were 6 cardiovascular-related deaths in the sitagliptin group compared to 3 in the placebo group. The exposure-adjusted incidence rate for cardiovascular-related death was 0.28 per 100-patient-years with sitagliptin and 0.15 with placebo (incidence rate ratio = 1.79 [95% CI: 0.44, 8.79]).

#### Sitagliptin vs. Sulphonylurea

Baseline characteristics were similar between treatment arms (data not shown). The cumulative patient exposure was approximately 1,269 patient-years in the sitagliptin group and 1,274 patient-years in the sulphonylurea group. In this three-study subanalysis, events were reported in all 3 studies. No patients in the sitagliptin group compared to 11 in the sulphonylurea group experienced a MACE-related event. The exposure-adjusted incidence rate was 0.00 per 100 patient-years with sitagliptin and 0.86 with sulphonylurea (adjusted incidence rate ratio = 0.00 [95% CI: 0.00, 0.31], reflective of no events in the sitagliptin group). There were no cardiovascular-related deaths in the sitagliptin group compared to 5 in the sulphonylurea group. The exposure-adjusted incidence rate for cardiovascular-related death was 0.00 per 100-patient-years with sitagliptin and 0.39 with sulphonylurea (adjusted incidence rate ratio = 0.00 [95% CI: 0.00, 0.81]). In the sensitivity analysis that included all blinded exposure to sitagliptin 100 mg or a sulphonylurea (n = 1,897 on sitagliptin, n = 1,389 on a sulphonylurea), the cumulative patient exposure was 2,044 patient-years for the sitagliptin group and 1,486 patient-years for the sulphonylurea group. A total of 24 patients had at least one MACE-related event reported, with 11 in the sitagliptin group and 13 in the sulphonylurea group. The exposure-adjusted incidence rate was 0.54 per 100 patient-years in the sitagliptin group and 0.87 in the non-exposed group (adjusted incidence rate ratio = 0.41 [95% CI: 0.17, 0.96]).

In sensitivity analyses for each of the cohorts evaluated, nearly identical adjusted incidence rate ratios were found for those in the MACE and death analyses using the Mantel-Haenszel method and the adjusted hazard ratios from Cox regression (data not shown).

## Discussion

In this pooled analysis of 14,611 patients from 25 clinical studies, treatment with sitagliptin was not associated with an increased risk of cardiovascular events relative to the control treatments used in the studies (both active and placebo). The present results extend and confirm the previously reported cardiovascular safety findings with sitagliptin [16]. To control for the confounding effects of pooling active comparators, the present analysis also examined the effects of sitagliptin versus placebo on cardiovascular safety and found no increased risk of cardiovascular events with sitagliptin. Furthermore, cardiovascular-related analyses with other DPP-4 inhibitors support the present findings of no evidence of cardiovascular harm [17-20]. In these other analyses, there also tended to be a numerical reduction in risk of cardiovascular events with DPP-4 inhibitors relative to non-exposure to DPP-4 inhibitors. Recently, when Monami et al. [33] combined the results of randomised clinical trials for DPP-4 inhibitors in a meta-analysis, a significant reduction in risk for MACE-related events was found with DPP-4 inhibitors. These findings need to be confirmed in prospective studies appropriately designed to assess cardiovascular outcomes. For sitagliptin, a placebo-controlled study assessing cardiovascular outcomes is currently underway in patients at increased risk for cardiovascular events (Clinicaltrials.gov: NCT00790205; [34]).

In a subanalysis of three studies comparing treatment with sitagliptin to sulphonylureas in patients with type 2 diabetes mellitus, a lower incidence and risk of MACE-related events was observed with sitagliptin compared to sulphonylurea. In the sensitivity analysis including all blinded exposure to sitagliptin or sulphonylurea and more reported events overall, a similar trend was observed. A lower incidence of cardiovascular events was also reported with linagliptin compared to glimepiride in a two-year trial [35]. The comparison between linagliptin and glimepiride is being further assessed in a long-term cardiovascular outcomes trial (Clinicaltrial.gov: NCT 01243424). These observations could reflect a deleterious effect of sulphonylureas, a beneficial effect of DPP-4 inhibitors, or a combination of both. While DPP-4 inhibitors and sulphonylureas both enhance insulin secretion, there are differences in their mechanisms of action, which have the potential for impacting cardiovascular safety.

Sulphonylureas bind to the SUR subunit (subtype SUR1) of the potassium ATP (K_ATP_) channel in the beta cell membrane and by inhibiting K_ATP_ channel activity, increase insulin release irrespective of ambient glucose concentrations [36]. Incretin-mediated enhancement of insulin release, in contrast, is glucose-dependent [15]. As a result of the mechanisms of action, hypoglycaemia is a common side effect with sulphonylurea therapy [37], whereas rates of hypoglycaemia with incretin-based therapies are not increased relative to placebo [38]. For example, in the three studies used for the present pooled analysis, there was a 3- to 6-fold increase in the incidence of symptomatic hypoglycaemia with sulphonylurea compared with sitagliptin [27-30]. Hypoglycaemia is associated with ischaemic complications in diabetic subjects [39]. Additionally, sulphonylureas bind to the SUR receptor (subtype SUR2) on cardiac myocytes and on endothelial cells, and thus may have direct effects on cardiovascular function [40]. Sulphonylureas have also been shown to abolish the beneficial effects of ischaemic preconditioning, a protective mechanism whereby a brief period of ischaemia is cardioprotective for subsequent periods of prolonged ischaemia [41,42].

Sulphonylureas are widely used as pharmacologic therapy in patients with type 2 diabetes mellitus. A concern regarding the potential for sulphonylurea-induced cardiovascular toxicity was first raised as a result of the University Group Diabetes Program (UGDP) study, in which an increase in mortality was observed with tolbutamide compared to both insulin and to placebo [43]. While controversy ensued regarding the validity of these findings [44,45] and their applicability to other drugs in this class, a warning regarding the increased risk of cardiovascular mortality was introduced and remains in the product information for all sulphonylureas marketed in the United States. Long-term prospective clinical studies have not reported an increased risk of cardiovascular events or mortality with sulphonylurea relative to other treatments in patients with recently- or newly-diagnosed type 2 diabetes mellitus [46,47]. However, many, but not all, observational studies have found an association between sulphonylurea use and an increase in cardiovascular events or mortality [21-25]. Of note, metformin was a main comparator in these observational studies and any differences could be attributed to the cardiovascular benefit of metformin [48] rather than a risk related to sulphonylurea. The combination of metformin and sulphonylurea has been associated with an increased risk of death in a sub-study of UKPDS 34 [48] or the composite endpoint of hospitalization for cardiovascular disease or mortality in a meta-analysis of observational studies [49]. These results have not been confirmed in a randomized, prospective clinical study.

In contrast to the data suggesting a detrimental effect of sulphonylureas on cardiovascular outcomes, preclinical and clinical mechanistic studies have suggested potential benefits of incretins and incretin-based therapies, such as DPP-4 inhibitors or GLP-1 agonists, on cardiovascular function and outcomes [50,51]. In animals, GLP-1 infusion protected the rat myocardium against ischaemia reperfusion injury [52,53]. Genetically-modified mice that are lacking the DPP-4 enzyme had improved survival post-myocardial infarction; similar protection was observed in normal mice treated with sitagliptin [54,55]. In humans, GLP-1 infusion enhanced endothelial function, as measured by forearm blood flow in response to acetylcholine; interestingly, coadministration of the sulphonylurea glyburide, but not glimepiride, abolished GLP-1 induced augmentation of forearm blood flow [56]. GLP-1 infusion also improved left ventricular ejection and contractile function in patients with acute myocardial infarction [57]. In a study of patients with coronary artery disease awaiting revascularisation, sitagliptin treatment was associated with improvement in left ventricular performance in response to dobutamine-induced stress and with mitigation of post-ischaemic stunning [58].

While the potential benefits of DPP-4 inhibition have been primarily attributed to the enhancement of GLP-1 activity, other endogenous substrates of DPP-4 could be relevant to cardiovascular function and outcomes. Treatment with sitagliptin increased plasma levels of stromal-derived factor-1α (SDF-1α) and circulating endothelial progenitor cells, while also reducing plasma monocyte chemoattractant protein-1, a proinflammatory chemokine, in patients with type 2 diabetes mellitus [59]. SDF-1α, a chemokine, attracts stem cells to ischaemic sites and enhances post-ischaemia angiogenesis [60]. Zaruba et al. [61] found that, in mice in which myocardial infarction was induced via surgical ligation of the left anterior descending artery, coadministration of granulocyte-colony-stimulating factor (GCSF; to stimulate stem cell mobilisation) with a DPP-4 inhibitor resulted in increased myocardial homing of circulating CXCR-4+ stem cells, reduced cardiac remodeling, and improved heart function and survival. A clinical trial assessing the safety and efficacy of sitagliptin in combination with GCSF in patients with acute myocardial infarction is currently underway [62].

The present results should be interpreted with caution because of the *post hoc* nature of this analysis and the clinical studies were not specifically designed to assess cardiovascular outcomes. Additionally, the case definition used in the analysis was based on reports of adverse events that matched MACE-related terms using the Medical Dictionary for Regulatory Activities and on cardiovascular-related deaths, rather than on the results of a formal process of adjudication of reported events. Any potential impact of rosiglitazone on the present findings was likely minimal, as rosiglitazone therapy was included in only two of the twenty-five studies. In one study in which rosiglitazone was a comparator agent, there were no MACE-related events reported. In the other study that had three MACE-related events, rosiglitazone and metformin were background therapy in both the sitagliptin and non-exposed groups.

The strengths of this analysis include the pooling of data from randomized controlled trials, the use of patient-level data, and a large sample size. The potential influence of baseline characteristics on outcomes was controlled by using studies in which patients were randomly assigned to treatment groups. Lastly, numerous supporting analyses confirmed the primary findings.

In summary, these analyses suggest that treatment with sitagliptin does not increase cardiovascular risk in patients with type 2 diabetes mellitus. In a subanalysis, a higher rate of cardiovascular morbidity and mortality was associated with sulphonylurea therapy relative to sitagliptin. Whether this observation is related to a deleterious effect of sulphonylurea therapy, a protective effect of sitagliptin, or a combination of the two is unknown at this time and will require future research.

## Appendix I

This analysis used a pooled population (N = 14,611) drawn from all 25 multicenter, U.S. or multinational, double-blind, parallel-group studies conducted by Merck & Co., Inc., in which patients were randomised to receive sitagliptin 100 mg/day (n = 7,726) or a comparator (n = 6,885) for at least 12 weeks and up to 2 years (the duration of the longest studies) and for which results were available as of December 1, 2011. Specific studies and treatment arms are listed in Table 2.

**Table 2:** Studies and treatment arms included in pooled analysis

**Study**	**Study design**	**Sitagliptin 100 mg/day group**^**§**^**(N = 7195)**	**n**	**Non-exposed group (N = 6267)**^**§**^	**n**	Reference*
**P010:** twice-daily dose-range finding	106-week active-controlled period	-Sitagliptin 50 mg b.i.d. switched to sitagliptin 100 mg q.d.	122	-Glipizide	123	[27]^†^
**P014:** once-daily dose-range finding	12-week placebo-controlled period and 94-week active-controlled period	-Sitagliptin 100 mg q.d.	110	-Placebo (12 weeks) switched to metformin (94 weeks)	111	[63]^††^
		-Sitagliptin 50 mg b.i.d. switched to sitagliptin 100 mg q.d.	111			
**P019:** placebo-controlled add-on to pioglitazone study	24-week placebo-controlled period	-Sitagliptin 100 mg q.d.	175	-Placebo	178	[64]^††^
**P020:** placebo-controlled add-on to metformin study	24-week placebo-controlled period and 80-week active-controlled period	-Sitagliptin 100 mg q.d.	464	-Placebo (24 weeks) switched to glipizide	237	[65]^††^
**P021:** placebo-controlled monotherapy study	24-week placebo-controlled period	-Sitagliptin 100 mg q.d.	238	-Placebo	253	[66]^††^
**P023:** placebo-controlled monotherapy study	18-week placebo-controlled period and 36-week active-controlled period	-Sitagliptin 100 mg q.d.	205	-Placebo (18 weeks) switched to pioglitazone (36 weeks)	110	[67]^††^
**P024:** active-controlled add-on to metformin study	104-week active-controlled period	-Sitagliptin 100 mg q.d.	588	-Glipizide	584	[28,29]^†^
**P035:** placebo-controlled add-on to glimepiride, alone or in combination with metformin study	24-week placebo-controlled period and 30-week active-controlled period	-Sitagliptin 100 mg q.d.	222	-Placebo (24 weeks) switched to pioglitazone (30 weeks)	219	[68]^††^
**P036:** placebo- and active-controlled study of initial combination use of sitagliptin and metformin	24-week placebo-controlled period; 80-week active-controlled period	-Sitagliptin 100 mg q.d.	179	-Placebo (24 weeks) switched to metformin (80 weeks)	176	[69,70,71]^††^
		-Sitagliptin 50 mg b.i.d. + metformin 500 mg b.i.d.	190	-Metformin 500 mg b.i.d.	182	
		-Sitagliptin 50 mg b.i.d. + metformin 1000 mg b.i.d.	182	-Metformin 1000 mg b.i.d.	182	
**P040:** placebo-controlled monotherapy study	18-week placebo-controlled period	-Sitagliptin 100 mg q.d.	352	-Placebo	178	[72]^††^
**P047:** placebo-controlled monotherapy study in elderly patients	24-week placebo-controlled period	-Sitagliptin 100 mg q.d.	91	-Placebo	92	[73]^††^
**P049:** active-controlled monotherapy study	24-week active-controlled period	-Sitagliptin 100 mg q.d.	528	-Metformin	522	[74]
**P051:** placebo-controlled add-on to insulin, alone or in combination with metformin study	24-week placebo-controlled period	-Sitagliptin 100 mg q.d.	322	-Placebo	319	[75]^††^
**P052:** placebo-controlled add-on to metformin and rosiglitazone study	54-week placebo-controlled period	-Sitagliptin 100 mg q.d.	170	-Placebo	92	[76]^††^
**P053:** placebo-controlled add-on to metformin study	30-week placebo-controlled period	-Sitagliptin 100 mg q.d.	96	-Placebo	94	[77]^††^
**P061:** placebo- and active-controlled mechanism of action factorial study	12-week placebo-controlled period	-Sitagliptin 100 mg q.d.	52	-Pioglitazone	54	[78]^††^
		-Sitagliptin 100 mg q.d. + pioglitazone	52	-Placebo	53	
**P064:** active-controlled study of initial combination use of sitagliptin and pioglitazone	54-week active-controlled period	-Sitagliptin 100 mg q.d. + pioglitazone	261	-Pioglitazone	259	[79,80]^††^
**P066:** active-controlled study of combination use of sitagliptin/metformin FDC	32-week active-controlled period	-Sitagliptin 50 mg + metformin 1000 mg b.i.d. (FDC)	261	-Pioglitazone 45 mg q.d.	256	[81]
**P068:** active-controlled study of sitagliptin and combination use of sitagliptin/metformin FDC	40-week active-controlled period	-Sitagliptin 100 mg q.d. switched to sitagliptin 50 mg + metformin 1000 mg b.i.d. (FDC)	244	-Pioglitazone 15 mg q.d. titrated up to 45 mg q.d.	247	[82]
**P074:** placebo-controlled add-on to metformin study	24-week placebo-controlled period	-Sitagliptin 100 mg q.d.	197	-Placebo	198	[83]^††^
**P079:** active-controlled study of initial combination use of sitagliptin/metformin FDC	44-week active-controlled period	-Sitagliptin 50 mg + metformin 1000 mg b.i.d. (FDC)	625	-Metformin 1000 mg b.i.d. (FDC)	621	[84,85]^††^
**P102:** active-controlled study of initial combination use of sitagliptin and pioglitazone	54-week active-controlled period	-Sitagliptin 100 mg q.d.	231			[86]^††^
		-Sitagliptin 50 mg b.i.d. + pioglitazone 15 mg q.d.	230	-Pioglitazone 15 mg q.d.	230	
		-Sitagliptin 50 mg b.i.d. + pioglitazone 30 mg q.d.	231	-Pioglitazone 30 mg q.d.	233	
		-Sitagliptin 50 mg b.i.d. + pioglitazone 45 mg q.d.	230	-Pioglitazone 45 mg q.d.	230	
**P128:** placebo-controlled add-on to metformin and pioglitazone study	26-week placebo-controlled period	-Sitagliptin 100 mg q.d.	157	-Placebo	156	[87]^††^
**P801:** placebo- and active-controlled add-on to metformin study	18-week placebo-controlled period	-Sitagliptin 100 mg q.d.	94	-Rosiglitazone 8 mg q.d.	87	[88]^††^
				-Placebo	91	
**P803:** active-controlled add-on to metformin study	30-week active-controlled period	-Sitagliptin 100 mg q.d.	516	-Glimepiride	518	[30]^†^

* References are for the initial phases of the studies that had extension or continuation phases, unless a reference is provided for the results beyond the initial phase.

^**§**^ This column reflects the blinded treatment(s) to which patients were randomised. For studies identified in column 1 as "add-on" studies, all patients also received the active therapy indicated in column 1 (open-label).

^†^ Studies included in the primary Sitagliptin vs. Sulphonylurea comparison.

^††^ Studies included in the primary Sitagliptin vs. Placebo comparison. This comparison included studies where patients were randomised to sitagliptin or placebo as monotherapy (e.g., P021) or as add-on therapy (e.g., P019), as well as studies where patients were randomised to sitagliptin + active agent or active agent (e.g., P102). The control groups of studies in the latter category included a sitagliptin-matched placebo for purposes of blinding. For studies where the placebo control group switched to active therapy at a post-randomisation time point (e.g., P020), only the placebo-controlled portion of the study was included.

q.d. = once daily; b.i.d. = twice daily; FDC = fixed-dose combination tablet.

## Appendix II: Definition of major adverse cardiovascular events (MACE)

Any adverse event with a Medical Dictionary for Regulatory Activities (MedDRA, version 14.1) term in the following list (Table 3) was classified as MACE. Asterisks indicate the MedDRA terms that were reported in the present analyses.

**Table 3:** MACE terms

Acute myocardial infarction*	Haemorrhagic cerebral infarction
Basal ganglia infarction	Haemorrhagic stroke*
Basilar artery thrombosis	Haemorrhagic transformation stroke
Brain stem infarction*	Ischaemic cerebral infarction
Brain stem stroke	Ischaemic stroke*
Brain stem thrombosis	Lacunar infarction*
Carotid arterial embolus	Lateral medullary syndrome
Carotid artery thrombosis	Moyamoya disease
Cerebellar artery thrombosis	Myocardial infarction*
Cerebellar embolism	Papillary muscle infarction
Cerebellar infarction*	Post procedural myocardial infarction
Cerebral artery embolism	Post procedural stroke
Cerebral artery thrombosis	Silent myocardial infarction*
Cerebral infarction*	Stroke in evolution
Cerebral thrombosis	Sudden cardiac death*
Cerebrovascular accident*	Thalamic infarction*
Coronary artery thrombosis	Thrombotic cerebral infarction
Coronary bypass thrombosis	Thrombotic stroke
Embolic cerebral infarction	Wallenberg syndrome
Embolic stroke	

Additionally, all deaths determined to be potentially cardiovascular-related (based on blinded clinical review) were classified as MACE. The reported MedDRA terms for the MACE-related events classified as cardiovascular (CV) deaths were as follows (Table 4):

**Table 4:** MACE-related cardiovascular death terms

Acute myocardial infarction	Fall
Cardio-respiratory arrest	Haemorrhagic stroke
Cerebral ischaemia	Ischaemic stroke
Coronary artery disease	Myocardial infarction
Coronary artery insufficiency	Myocardial ischaemia
Death	Sudden cardiac death
Drowning	

## Competing interests

All authors are employees of Merck Sharp & Dohme Corp., a subsidiary of Merck & Co., Inc., Whitehouse Station, NJ, the manufacturer of sitagliptin and may have stock or stock options in the company.

## Authors' contributions

SSE, GTG, DS, KDK, and BJG conceived the design for the analyses. GTG performed the statistical analyses. All authors were involved in the interpretation of the analyses. All authors were involved in drafting the manuscript or revising it critically for important intellectual content. All authors approved the final manuscript.

## Funding

All studies and analyses described in this review were funded by Merck Sharp & Dohme Corp., a subsidiary of Merck & Co., Inc., Whitehouse Station, NJ.
